# Megalin dependent urinary cystatin C excretion in ischemic kidney injury in rats

**DOI:** 10.1371/journal.pone.0178796

**Published:** 2017-06-02

**Authors:** Danny Jensen, Casper Kierulf-Lassen, Marie Louise Vindvad Kristensen, Rikke Nørregaard, Kathrin Weyer, Rikke Nielsen, Erik Ilsø Christensen, Henrik Birn

**Affiliations:** 1 Department of Biomedicine, Institute of Health, Aarhus University, Aarhus, Denmark; 2 Department of Renal Medicine, Aarhus University Hospital, Aarhus, Denmark; 3 Institute of Clinical Medicine, Aarhus University, Aarhus, Denmark; George Washington University School of Medicine and Health Sciences, UNITED STATES

## Abstract

**Background:**

Cystatin C, a marker of kidney injury, is freely filtered in the glomeruli and reabsorbed by the proximal tubules. Megalin and cubilin are endocytic receptors essential for reabsorption of most filtered proteins. This study examines the role of these receptors for the uptake and excretion of cystatin C and explores the effect of renal ischemia/reperfusion injury on renal cystatin C uptake and excretion in a rat model.

**Methods:**

Binding of cystatin C to megalin and cubilin was analyzed by surface plasmon resonance analysis. ELISA and/or immunoblotting and immunohistochemistry were used to study the urinary excretion and tubular uptake of endogenous cystatin C in mice. Furthermore, renal uptake and urinary excretion of cystatin C was investigated in rats exposed to ischemia/reperfusion injury.

**Results:**

A high affinity binding of cystatin C to megalin and cubilin was identified. Megalin deficient mice revealed an increased urinary excretion of cystatin C associated with defective uptake by endocytosis. In rats exposed to ischemia/reperfusion injury urinary cystatin C excretion was increased and associated with a focal decrease in proximal tubule endocytosis with no apparent change in megalin expression.

**Conclusions:**

Megalin is essential for the normal tubular recovery of endogenous cystatin C. The increase in urinary cystatin C excretion after ischemia/reperfusion injury is associated with decreased tubular uptake but not with reduced megalin expression.

## Introduction

Cystatin C (cysC) is an endogenous protease inhibitor (Molecular weight ~ 13.4 kDa) produced at a constant rate in all nucleated cells[1]. It is freely filtered and thus the plasma concentration of cysC is dependent on the glomerular filtration rate (GFR) and cysC production rate. In healthy individuals essentially no cysC is excreted in the urine [2] due to effective reabsorption and degradation of filtered cysC in the proximal tubule (PT) [3]. Urinary cysC excretion (U-cysC) has been studied as a marker of kidney injury, most likely reflecting tubular dysfunction [4]. Proximal tubule (PT) disorder, such as tenofovir induced tubular dysfunction, has been associated with an increased U-cysC [5]. U-cysC predicts the development of acute kidney injury (AKI) [6] and subsequent renal failure [7]. In renal transplantation, levels of U-cysC, as early as 6 hours post-operative, are associated with late graft function [8]. In chronic kidney disease (CKD) U-cysC correlates with GFR, and it has been demonstrated that patients with GFR < 30 mL/min have a higher U-cysC excretion when compared to patients with GFR > 70 mL/min [9, 10]. In a diabetic rat model increased U-cysC preceded and predicted the development of diabetic nephropathy [11] and in humans with diabetes increased U-cysC was associated with a greater risk of declining eGFR [12]. This suggests that U-cysC, as a marker of early tubular dysfunction, may precede the decline in GFR, and serve as a marker of progression in CKD.

The multi-ligand endocytic receptors megalin and cubilin are heavily expressed in the PT brush border membrane and essential for the reabsorption of a large number of filtered proteins in the PT [13–15]. Endocytosis of cubilin and cubilin ligands in the PT is dependent on the interaction with megalin [16] and the two proteins act in concert to mediate tubular recovery of a number of established urinary biomarkers such as albumin, vitamin D-binding protein (DBP), retinol binding protein (RBP), alpha-1-microglobulin, and neutrophil gelatinase-associated lipocalin (NGAL) [17–22]. Injected exogenous cysC binds to megalin and is reabsorbed by a megalin dependent process in the PT [23]; however, the molecular mechanisms regulating the reabsorption of endogenous cysC have not been fully characterized, and while it has been hypothesized that in both AKI and CKD the increased urinary excretion of cysC (and other biomarkers) is due to decreased reabsorption of cysC in the PT [24], the exact mechanism and role of megalin and cubilin has not been fully characterized.

To study the importance of megalin and cubilin for the normal tubular reabsorption of endogenous cysC, we analyzed the *in vitro* binding of cysC to these receptors and examined renal handling of cysC *in vivo* in mice with conditional kidney specific knockout of the megalin and/or cubilin genes. To further characterize the mechanism of increased U-cysC in AKI we evaluated the effect of ischemia/reperfusion (I/R) injury in rats on megalin expression, U-cysC, and tubular cysC uptake.

## Materials and methods

### Materials

Affinity purified human cysC was purchased from BioVender R&D (Brno, Czech Republic) Cat. No: RD172009100. Primary polyclonal goat anti-mouse cysC antibodies were obtained from R&D Systems (Abingdon, United Kingdom) catalog number AF1238. Primary polyclonal rabbit anti mouse megalin antibody was a gift from E. de Heer, Dept. of pathology, at University of Leiden (Leiden, Holland). Primary polyclonal rabbit anti mouse cubilin antibody was obtained from P. Verroust, INSERM U64 (Paris, France). The secondary antibodies included Alexa Fluor® 488 donkey anti-rabbit IgG and Alexa Fluor® 568 donkey anti-goat IgG (Thermo Fisher Scientific inc., Waltham, USA). Rat U-cysC and mouse plasma cysC were measured by ELISA as specified by the supplier (Quantikine® ELISA, R&D Systems (Abingdon, United Kingdom), Cat. No. MSCTC0).

### Animal models

All animal experiments were performed in accordance with provisions for the animal care licenses provided by the Danish National Animal Experiments Inspectorate, and approved by the ethics comity of the Danish National Animal Experiments Inspectorate. All animals had free access to a standard rodent diet and water, and were kept in a 12:12 hour light-dark cycle. Urine was collected for 24 hours in metabolic cages. Rats used for perfusion fixation were sacrificed by intra peritoneal pentobarbital injection. Remaining rats were sacrificed by cervical dislocation at end of study. Mice used for preparation of sections were anesthetized by isoflouran, and perfusion-fixed while unconscious–the remainder of the mice were returned to their original cages.

#### Megalin and/or cubilin deficient mice

Cub ^-/-^/meg^-/-^ mice as well as cub ^-/-^ mice under control of the Wnt4 promoter were produced as previously described [25]. These mice have approx. 90% reduction in kidney PT megalin or cubilin expression [25]. Wildtype mice on a similar, mixed 129/C57 background were used as controls. Kidneys from 9 mice (3 cub ^-/-^ mice, 3 cub ^-/-^/meg^-/-^ mice, and 3 wildtype) were fixed by retrograde perfusion through the abdominal aorta with 2% paraformaldehyde in 0.1 M sodium cacodylate buffer, pH 7.4 for paraffin embedding and immunohistochemistry.

Urine was collected from 12 mice (4 cub ^-/-^ mice, 4 cub ^-/-^/meg^-/-^mice and 4 wildtype mice ranging from 55 to 106 weeks of age). 12 plasma samples (4 cub ^-/-^ mice, 4 cub ^-/-^/meg^-/-^mice and 4 wildtype mice) collected in another study were used to compare plasma cysC levels in the 3 phenotypes.

#### Unilateral nephrectomy followed by I/R injury in rats

Thirty-nine male, Wistar rats with a starting weight of 230–250 g were included in the study (16 sham operated and 23 I/R operated). All surgery was performed through an abdominal incision under anesthesia with sevoflurane. Animals were placed on a heating pad to maintain a rectal temperature of approx. 36°C. At onset of surgery and every 8 to12 hours for the first 24 hours after surgery, rats were injected with buprenorphine, 0.05 mg/kg subcutaneously (SC) after which buprenorphine (0.3 mg/ml) was supplied in the drinking water for 48 hours for post-operative pain relieve. Rats were allowed recovery from anesthesia before being returned to the cages.

The right kidney was removed 7 (+/- 1) days prior to I/R injury. I/R injury was induced, by clamping of the left renal artery for 37 minutes using a non-traumatic clamp, after which the clamp was removed. The obstruction of blood flow as well as reperfusion was confirmed by color changes of the kidney. To avoid arterial spasm lidocaine was applied to the artery around the clamp prior to removal. The sham-operated controls underwent same procedure except for the arterial clamping. During nephrectomy and I/R surgery, rats were injected SC with 4 mL of isotonic saline. This was repeated day 1 after surgery. Twenty-four hour urine samples (n = 31) were collected 1 day prior to as well as two and six days after I/R or sham operation. Eight rats, 4 sham and 4 I/R operated, were sacrificed at day 3 after surgery for perfusion fixation of the left kidney as described earlier.

### Surface plasmon resonance analysis

Human cysC was dissolved in running buffer (CaHBS: 10 mM HEPES, 150 mM NaCl, 1.5 mM CaCl2, 1 mM EGTA, pH 7.4) at 7 different concentrations (20 nM, 50 nM, 100 nM, 200 nM, 300 nM, 400 nM and 500 nM) and analyzed for binding to purified, immobilized rat megalin and cubilin [26] on a Biacore® 3000 surface plasmon resonance apparatus (GE healthcare, Pittsburgh, USA). In order to specifically inhibit cysC binding to megalin and cubilin the chip was pre-saturated with receptor associated protein (RAP) (5 μM), a well-known inhibitor of ligand binding to megalin. To identify calcium dependent binding 20 mM of EDTA was added to the running buffer with cysC at a concentration of 150 and 300 nM, respectively. Kinetic parameters were determined by BIAevaluation 4.1 software (GE healthcare, Pittsburgh, USA) using a Langmuir 1:1 binding-model and global fitting.

### Immunoblotting

Two μL of urine sample, diluted according to urine output, were mixed with 4X Laemmli buffer, heated to 95°C for 5 min, loaded onto a 16% gel, and separated by standard SDS-PAGE. Kaleidoscope™ Precision Plus Protein™ Standards (Hercules, USA) was used as size marker. Protein was transferred to polyvinylidene difluoride membrane using the iBlotTM Dry Blotting System (Invitrogen, Thermo Fisher Scientific inc., Waltham, USA), fixed with Odyssey® (LI-COR, Inc., Lincoln, USA) blocking buffer (BB) diluted 1:1 with 0.1 M phosphate buffered saline (PBS), then incubated with 6 mL goat anti-mouse cysC antibody (0.1 μg/mL) in 1:1 BB/PBS (0.1 M) solution and left overnight at 4°C. Following wash the membranes were incubated for 1 hour with the relevant secondary antibody in PBS. Proteins were detected using OdysseyTM infrared imager (LI-COR, Inc., Lincoln,USA).

### Immunohistochemistry

Kidney tissue was fixed as described earlier, dehydrated and embedded in paraffin. For visualization of cysC, megalin, and cubilin in the kidney cortex, 2 μm paraffin sections were cut and rehydrated using standard methods. Sections were labeled using anti megalin (1:400/1:800), anti cysC (1:60), and/or anti cubilin (1:4000) primary antibodies over night at 4°C followed by incubation with relevant secondary antibodies for 60 minutes at room temperature. 0.01 M PBS containing 0.1% bovine serum albumin, 0.3% Triton X-100 adjusted to pH 7.4 was used for antibody dilution. Control labeling experiments involved incubation with no primary antibody or each of the three primary antibodies individually followed by the secondary antibodies. No cross reactivity or unspecific secondary binding was detected. Images were recorded using a Leica TCS SL Spectral Confocal Microscope (TM Leica Microsystems, Wetzlar, Germany).

### Statistical analysis

Normal distribution of data was obtained after transformation to a log10 scale and normality was confirmed using qq-plots and histograms. Paired and unpaired students t-test was used when appropriate to compare data. A p-value < 0.05 was considered significant. The STATA 12.1 (StataCorp LP, College Station, USA) software was used for analysis.

## Results

### CysC binds both to purified megalin and cubilin

**Fig 1:**
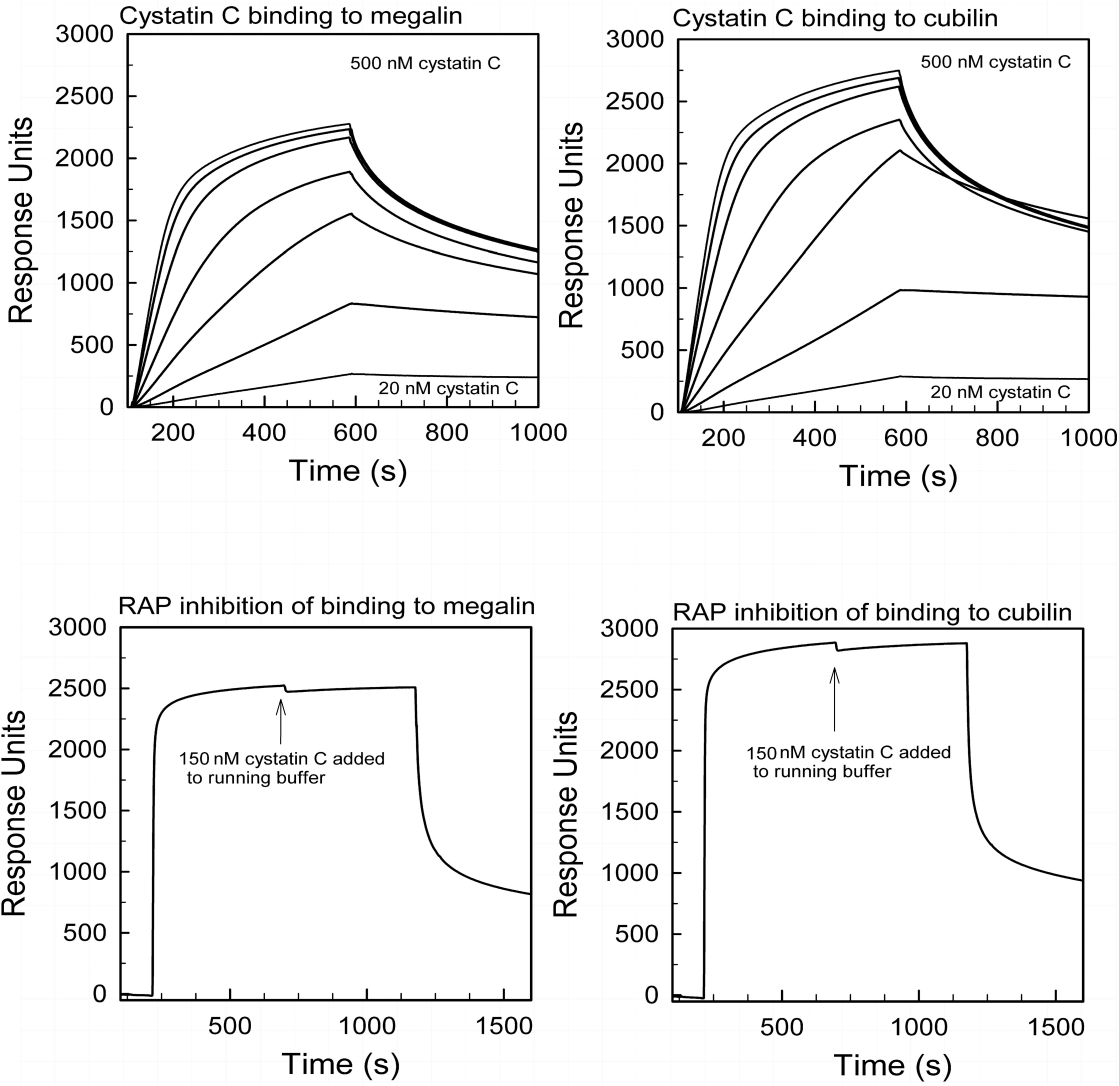
Cystatin C binding to megalin and cubilin in surface plasmon analysis. Surface plasmon resonance analysis showing binding of purified human cysC to immobilized megalin (A) and cubilin (B) at dilutions ranging from 20 nM to 500 nM. The calculated Kd was approximately 32 nM for binding to megalin and 24 nM for binding to cubilin. In (C) and (D) the flow of cystatin C (150 nM) was preceded by binding of RAP (5 μM) essentially abolishing the binding to megalin (C) and cubilin (D).

Surface plasmon resonance analysis revealed a high affinity binding of cysC to megalin (Kd ~ 32 nM) and cubilin (Kd ~ 24 nM). Receptor associated protein (RAP), a known inhibitor of ligand binding to megalin, inhibited cysC binding to megalin and cubilin completely (Fig 1). The addition of 20 mM EDTA did not inhibit cysC binding to megalin or cubilin (data not shown).

### U-cysC excretion is increased in megalin/cubilin deficient mice and associated with decreased proximal tubule uptake

**Fig 2:**
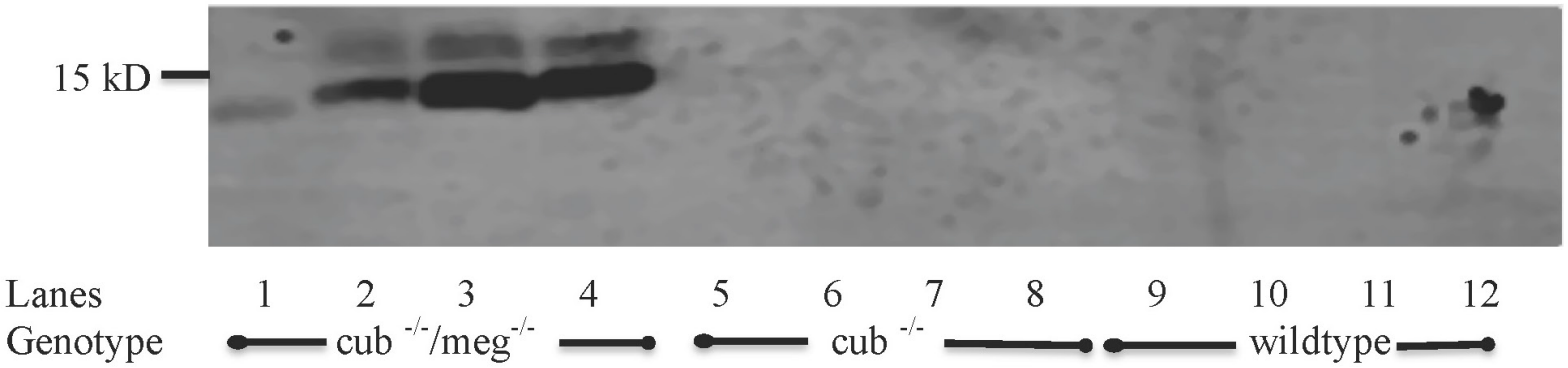
Urinary cystatin C excretion in mice. Western blot of urine samples, showing the presence of cysC in urine from cub ^-/-^/meg^-/-^ mice (lanes 1–4), cub ^-/-^ mice (lanes 5–8), and wildtype mice (lanes 9–12). CysC was identified in the urine from cub ^-/-^/meg^-/-^ mice only. The urines were loaded on the gel according to excretion rate.

Urinary excretion of cysC was identified as a single, approximately 13 kDa band in urine samples from cub ^-/-^/meg^-/-^ mice, but not cub ^-/-^ or wildtype mice (Fig 2).

**Fig 3:**
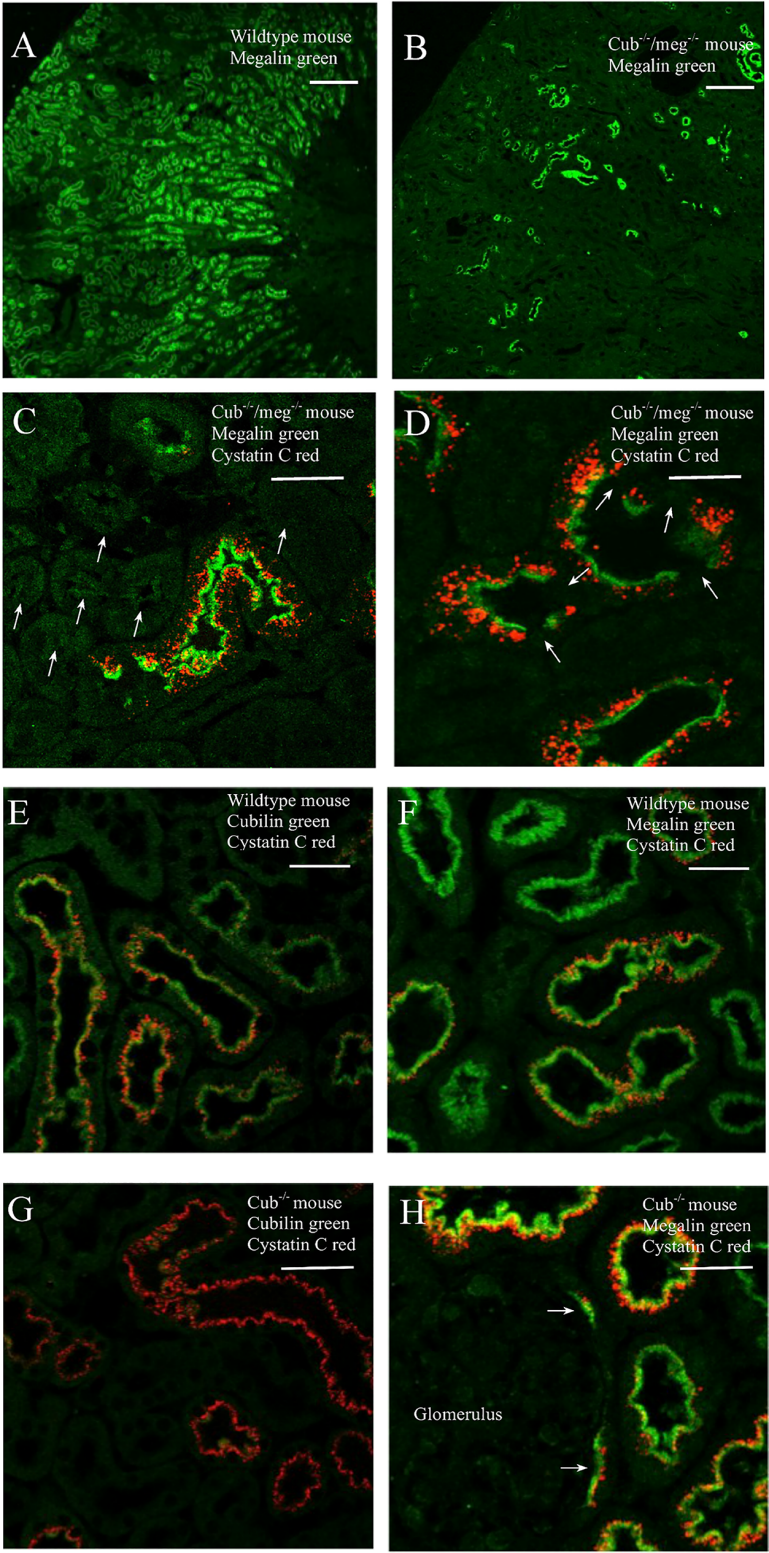
Renal cortical sections from mice. Immunohistochemistry was used to identify megalin, cubilin, and cysC in kidney cortical sections from wildtype (A, E and F), cub ^-/-^ (G and H) and cub ^-/-^/meg^-/-^ mice (B, C and D). In proximal tubule brush border of wildtype mice the expression of cubilin (green A) and megalin (green B) co-localized with vesicular accumulation of cysC (red A and B). In Cub ^-/-^ mouse the labeling for cysC appeared similar to wildtype despite the absence of immuno-detectable cubilin (G). Megalin labeling appeared normal in Cub ^-/-^ (H). Note that megalin is also expressed in proximal tubule cells forming part of Bowman’s capsule with corresponding cysC uptake (arrows in H). In cub^-/-^/meg^-/-^ mouse residual expression of megalin was observed only in a few mosaic tubular profiles (B and C (arrows indicating proximal tubules not expressing megalin)) when compared to wildtype (F) illustrating the high degree of conditional knockout. Uptake of cysC was observed only in the few proximal tubular cells with residual megalin expression selected in (C and D). Note that cells not expressing megalin do not reveal any cysC uptake (arrows in D). Scalebars in sections A, C, D, G and H correspond to 50 μm and scalebars in sections A and B correspond to 200 μm.

Immunohistochemistry revealed no uptake of cysC in megalin deficient PT cells (indicated by arrow in Fig 3C). A mosaic expression of megalin is noted in the PT of cub ^-/-^/meg^-/-^ mice with a few tubular profiles revealing residual expression of megalin (Fig 3C and 3D). These cells do show labeling for endogenous cysC (Fig 3D) and serve as an internal control. We observed vesicular labeling for cysC in wildtype PT cells consistent with endocytic uptake of filtered, endogenous cysC (Fig 3E and 3F). Similar labeling was observed in cubilin deficient cells (Fig 3G and 3H). Mean plasma levels of cysC were significantly higher in cub ^-/-^/meg^-/-^ -mice (620 (791; 472) ng/mL) than in cub ^-/-^ (457 (633; 315) ng/mL) and wildtype mice (433 (560; 326) ng/mL).

### U-CysC excretion is increased following I/R injury in rats, but is not associated with reduced megalin expression

A significant increase in U-cysC excretion was observed in rats exposed to I/R injury two days after the intervention when compared to baseline. A significant decrease of U-cysC in the sham group was observed at day six, when compared to baseline. U-cysC levels in I/R injury exposed rats, was significantly different (higher) from sham group at day two and six (Table 1).

**Table 1:** U-cysC excretion after I/R injury vs sham rats.

	Baseline			2 days post op.			6 days post op.		
	n	Cystatin C excretion		n	Cystatin C excretion		n	Cystatin C excretion	
		Median	Inter quartile range		Median	Inter quartile range		Median	Inter quartile range
**Sham**	8	78	73; 83	12	78	51; 83	6	66# ^(#p = 0,03)^	62; 69
**I/R**	18	91	64; 120	19	288*# ^(*#p = 0.000)^	161; 884	10	143* ^(*p = 0.04)^	94; 170

Rat U-cysC at baseline, two and six days after I/R injury or sham operation.

Significant differences from sham are marked with * (unpaired t-test).

Significant differences from baseline are marked with # (paired t-test).

Cystatin C excretion rates are given in microgram/day/kg rat.

**Fig 4:**
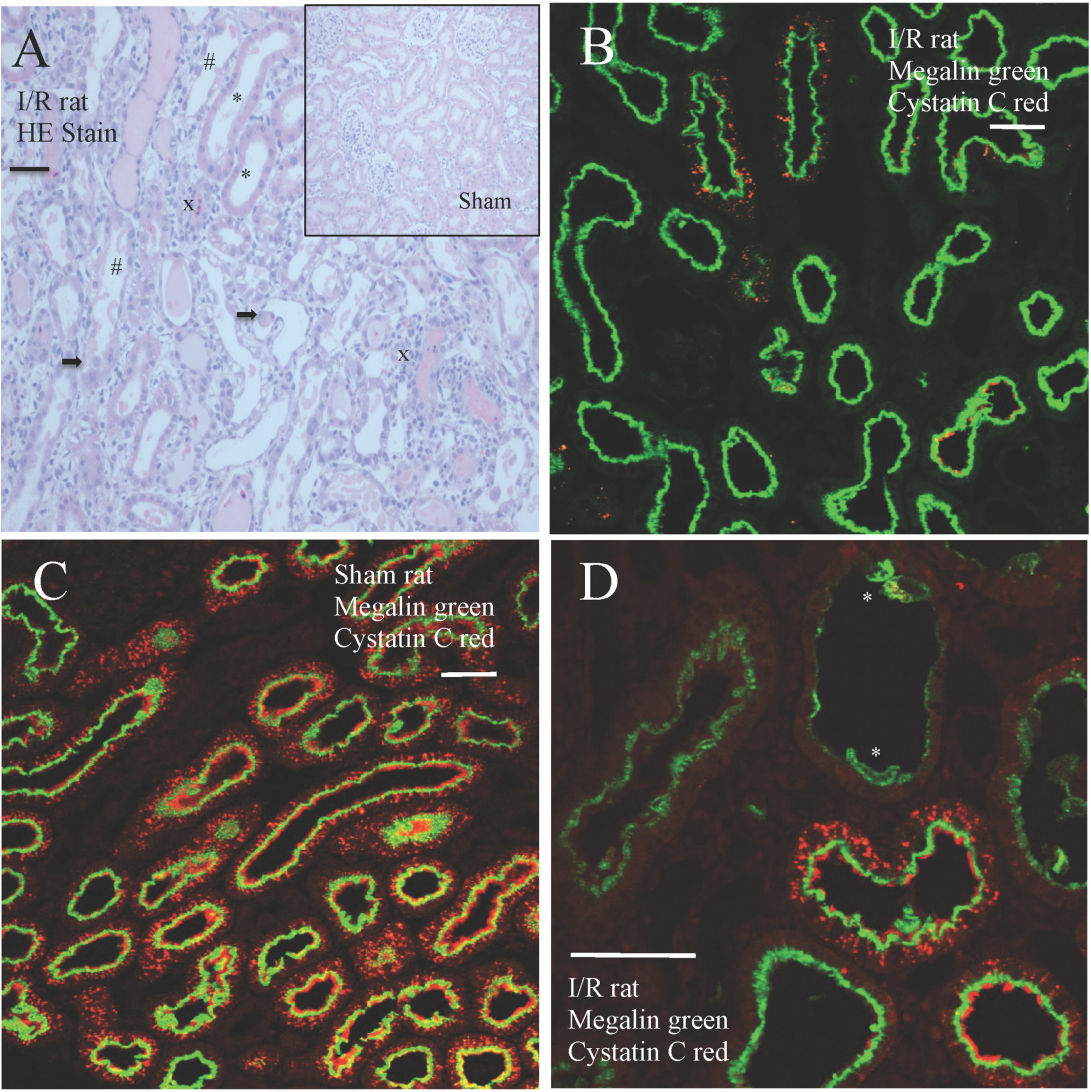
Renal cortical sections of rats exposed to I/R injury. HE stain of a cortical kidney section from I/R exposed rat (4A). Sham operated rat section is shown as insert in right corner for comparison. Evidence of acute tubular injury can be observed, consisting of tubular luminal distension (*), irregular nuclear size (#), interstitial inflammation (x) and loss of tubular cell adhesion to the basal membrane (arrow). Immunohistochemistry identified megalin (green) and cysC (red) in kidney cortical sections from rats two days after I/R injury (4B) or sham operation (4C). Normal, endocytic uptake of cysC was observed in most megalin expressing cells in sham operated rats (4C). In I/R operated rats, a clearly reduced, intracellular labeling for cysC was observed indicating a reduced endocytic uptake (4B), despite preserved expression of megalin in the proximal tubule brush border, Megalin, but not cysC, was identified in tubules with evidence of proximal tubule cell injury as indicated by *(4D). Scalebar = 50 μm.

In rats subjected to I/R injury morphological evidence of PT cell injury was evident. We observed cell detachment from the basal membrane, tubular dilation, and morphologic signs of acute tubular necrosis with interstitial hyper-cellularity, but also chromatin accumulation as sign of cell protein synthesis and cell regeneration (Fig 4A). Immunohistochemistry revealed reduced vesicular labeling for cysC in the I/R treated rats despite preserved expression of megalin in most PT cells (Fig 4B). This could suggest decreased turnover of endocytic vesicles, not reduced megalin expression *per se*, as the cause of increased U-cysC excretion in this model. In sham operated rats we observed a normal vesicular labeling for cysC co-localizing with megalin expressing PT cells, consistent with endocytic uptake of filtered, endogenous cysC (Fig 4C). Cubilin staining followed the same pattern as megalin (not shown).

## Discussion

This study demonstrates that the multi-ligand, endocytic receptor megalin is essential for the normal PT recovery of filtered, endogenous cysC. It also demonstrates that increased U-cysC excretion, as a marker of PT I/R injury, is the result of defective PT receptor mediated endocytosis, not reduced megalin expression in the PT. This is based on the following observations: 1) Megalin binds cysC with high affinity, which can be inhibited by RAP, 2) increased urinary excretion of endogenous cysC in megalin deficient mice is associated with abolished endocytic uptake in the PT cells, 3) U-cysC increases significantly following I/R injury, and finally 4) PT uptake of cysC, as reflected by an intracellular, vesicular labeling, is decreased in cells with evidence of injury despite the preserved expression of megalin.

The binding of cysC to megalin is consistent with findings of Kaseda et al. who examined renal handling of injected, exogenous cysC [23]. We observed an additional binding of cysC to cubilin; however, this binding did not appear to be essential for urinary excretion or tubular uptake as demonstrated by the normal tubular cysC handling in cubilin deficient mice. Similar binding characteristics have been demonstrated for other filtered megalin and cubilin ligands. Nykjaer et al. showed that DBP binds with strong affinity to megalin and cubilin when examined by surface plasmon resonance [27]; however, when urinary excretion of DBP was examined using cubilin deficient mice it was independent of cubilin [17]. In humans, evidence suggests that the reabsorption of DBP may at least in part depend on cubilin. Albumin also binds to both megalin [28] and cubilin. Unlike cysC, cubilin deficient mice reveal albuminuria even in the presence of functional megalin [25] probably due to the much higher affinity of albumin binding to cubilin when compared to megalin. In the absence of both megalin and cubilin the urinary excretion of albumin increases further [25] demonstrating the significance of the cubilin-megalin interaction. In this study, plasma levels of cysC observed in the double KO mice were significantly higher than the observed plasma cysC levels in the cubilin KO and the wildtype mice. This could be due to a decreased number of functional nephrons in the double KO mouse model, caused by the absence of megalin during nephro-genesis or podocyte function as suggested by Nielsen et al [29]. If this is the case, the total amount of cysC filtered, per nephron, in the double KO mice in steady state is higher than in the cubilin KO and wildtype mice. This increased per nephron cysC load in the double KO mice may contribute to the increased urinary excretion of cysC observed in the double KO mice. Never the less, we believe the total absence of cysC endocytosis in tubular cells not expressing megalin, to be the primary cause of the increased urinary cysC excretion in megalin KO mice, as observed in this study.

Ligand binding to megalin and cubilin is believed to be Ca^2+^-dependent, which has been demonstrated with many different ligands [23, 30–32]. In our study EDTA did not inhibit the *in vitro* binding of cysC to immobilized megalin or cubilin. This is consistent with the observation that FITC labeled cysC binding to purified rat brush-border membrane using a rapid filtration technique was reduced only 40% when increasing the EDTA concentration in the incubation buffer to 20 mM suggesting some Ca^2+^ independent binding of cysC to megalin [31]. Ca^2+^ independent binding to megalin has also been demonstrated for insulin [33].

A previous study of cisplatin induced AKI in rats [34] showed no changed in tissue content of cysC suggesting that this was due to a combination of decreased uptake and catabolism of cysC. In contrast, we observed a decrease in tubular cysC labeling suggesting decreased tubular uptake and no evidence of intracellular cysC accumulation. This suggests that the increase in U-CysC following I/R injury might be due to dysfunction of the endocytic process with no apparent change in megalin expression. We observed specific tubular damage with loss of cell polarity and adhesion to the tubular basal membrane and interstitial hyper-cellularity in our I/R rats. The PT cells in the segments S1 and S3 are very susceptible to I/R damage [35] due to their high metabolic rate and demand for ample oxygen resources which could explain these observations.

Several experimental studies using animal models of AKI [36], CKD [37], hypertensive [38] and diabetic nephropathy [39] have reported changes in megalin expression in the proximal tubule; however, few studies have addressed the endocytic function of megalin in kidney injury. When feeding rats large doses of L-Lysine Thelle et al [40] induced tubular proteinuria and abolished megalin dependent endocytosis in the PT. In their study, megalin and cubilin expression in the tubular brush border membrane was preserved. They concluded that the observed tubular proteinuria was, at least in part, due to dysfunctional vesicle trafficking–not just inhibition of ligand binding to megalin. We found cysC endocytosis to be dependent on binding to megalin; however, decreased megalin expression does not appear to be the major cause of increased U-cysC excretion in I/R injury. Thus, the urinary excretion of cysC may be a marker of megalin dependent endocytic function in the proximal tubule. Whether this will provide important pathophysiological information in acute or chronic kidney injury remains to be established.

## References

[1] Abrahamson M, Olafsson I, Palsdottir A, Ulvsback M, Lundwall A, Jensson O, et al Structure and expression of the human cystatin C gene. The Biochemical journal. 1990;268(2):287–94. Epub 1990/06/01. PubMed Central PMCID: PMC1131430. 2363674 10.1042/bj2680287 PMC1131430

[2] Uchida K, Gotoh A. Measurement of cystatin-C and creatinine in urine. Clinica chimica acta; international journal of clinical chemistry. 2002;323(1–2):121–8. Epub 2002/07/24. 12135813 10.1016/s0009-8981(02)00177-8

[3] Tenstad O, Roald AB, Grubb A, Aukland K. Renal handling of radiolabelled human cystatin C in the rat. Scandinavian journal of clinical and laboratory investigation. 1996;56(5):409–14. Epub 1996/08/01. doi: 10.3109/00365519609088795 8869663 10.3109/00365519609088795

[4] Herget-Rosenthal S, van Wijk JA, Brocker-Preuss M, Bokenkamp A. Increased urinary cystatin C reflects structural and functional renal tubular impairment independent of glomerular filtration rate. Clin Biochem. 2007;40(13–14):946–51. doi: 10.1016/j.clinbiochem.2007.04.013 17537416 10.1016/j.clinbiochem.2007.04.013

[5] Jaafar A, Seronie-Vivien S, Malard L, Massip P, Chatelut E, Tack I. Urinary cystatin C can improve the renal safety follow-up of tenofovir-treated patients. Aids. 2009;23(2):257–9. doi: 10.1097/QAD.0b013e328314e382 19098496 10.1097/QAD.0b013e328314e382

[6] Zhang Z, Lu B, Sheng X, Jin N. Cystatin C in prediction of acute kidney injury: a systemic review and meta-analysis. Am J Kidney Dis. 2011;58(3):356–65. Epub 2011/05/24. doi: 10.1053/j.ajkd.2011.02.389 21601330 10.1053/j.ajkd.2011.02.389

[7] Koyner JL, Vaidya VS, Bennett MR, Ma Q, Worcester E, Akhter SA, et al Urinary biomarkers in the clinical prognosis and early detection of acute kidney injury. Clinical journal of the American Society of Nephrology: CJASN. 2010;5(12):2154–65. Epub 2010/08/28. PubMed Central PMCID: PMC2994075. doi: 10.2215/CJN.00740110 20798258 10.2215/CJN.00740110 PMC2994075

[8] Snoeijs MG, van Bijnen A, Swennen E, Haenen GR, Roberts LJ 2nd, Christiaans MH, et al Tubular epithelial injury and inflammation after ischemia and reperfusion in human kidney transplantation. Annals of surgery. 2011;253(3):598–604. Epub 2011/01/21. doi: 10.1097/SLA.0b013e31820d9ae9 21248631 10.1097/SLA.0b013e31820d9ae9

[9] Donadio C. Serum and urinary markers of early impairment of GFR in chronic kidney disease patients: diagnostic accuracy of urinary beta-trace protein. American journal of physiology Renal physiology. 2010;299(6):F1407–23. Epub 2010/09/17. doi: 10.1152/ajprenal.00507.2009 20844024 10.1152/ajprenal.00507.2009

[10] Nakai K, Kikuchi M, Omori S, Saito K, Suwabe A. [Evaluation of urinary cystatin C as a marker of renal dysfunction]. Nihon Jinzo Gakkai shi. 2006;48(5):407–15. 16913462

[11] Togashi Y, Miyamoto Y. Urinary cystatin C as a biomarker for diabetic nephropathy and its immunohistochemical localization in kidney in Zucker diabetic fatty (ZDF) rats. Experimental and toxicologic pathology: official journal of the Gesellschaft fur Toxikologische Pathologie. 2013;65(5):615–22. Epub 2012/07/17. 22795897 10.1016/j.etp.2012.06.005

[12] Kim SS, Song SH, Kim IJ, Jeon YK, Kim BH, Kwak IS, et al Urinary cystatin C and tubular proteinuria predict progression of diabetic nephropathy. Diabetes care. 2013;36(3):656–61. Epub 2012/10/25. PubMed Central PMCID: PMC3579333. doi: 10.2337/dc12-0849 23093662 10.2337/dc12-0849 PMC3579333

[13] Christensen EI, Birn H. Megalin and cubilin: multifunctional endocytic receptors. Nature reviews Molecular cell biology. 2002;3(4):256–66. Epub 2002/05/08. doi: 10.1038/nrm778 11994745 10.1038/nrm778

[14] Seetharam B, Levine JS, Ramasamy M, Alpers DH. Purification, properties, and immunochemical localization of a receptor for intrinsic factor-cobalamin complex in the rat kidney. The Journal of biological chemistry. 1988;263(9):4443–9. Epub 1988/03/25. 2831230

[15] Moestrup SK, Kozyraki R, Kristiansen M, Kaysen JH, Rasmussen HH, Brault D, et al The intrinsic factor-vitamin B12 receptor and target of teratogenic antibodies is a megalin-binding peripheral membrane protein with homology to developmental proteins. The Journal of biological chemistry. 1998;273(9):5235–42. Epub 1998/03/28. 9478979 10.1074/jbc.273.9.5235

[16] Christensen EI, Birn H, Storm T, Weyer K, Nielsen R. Endocytic receptors in the renal proximal tubule. Physiology (Bethesda). 2012;27(4):223–36. Epub 2012/08/10. 22875453 10.1152/physiol.00022.2012

[17] Amsellem S, Gburek J, Hamard G, Nielsen R, Willnow TE, Devuyst O, et al Cubilin is essential for albumin reabsorption in the renal proximal tubule. Journal of the American Society of Nephrology: JASN. 2010;21(11):1859–67. Epub 2010/08/28. PubMed Central PMCID: PMC3014001. doi: 10.1681/ASN.2010050492 20798259 10.1681/ASN.2010050492 PMC3014001

[18] Kozyraki R, Fyfe J, Verroust PJ, Jacobsen C, Dautry-Varsat A, Gburek J, et al Megalin-dependent cubilin-mediated endocytosis is a major pathway for the apical uptake of transferrin in polarized epithelia. Proceedings of the National Academy of Sciences of the United States of America. 2001;98(22):12491–6. Epub 2001/10/19. PubMed Central PMCID: PMC60081. doi: 10.1073/pnas.211291398 11606717 10.1073/pnas.211291398 PMC60081

[19] Christensen EI, Moskaug JO, Vorum H, Jacobsen C, Gundersen TE, Nykjaer A, et al Evidence for an essential role of megalin in transepithelial transport of retinol. Journal of the American Society of Nephrology: JASN. 1999;10(4):685–95. Epub 1999/04/15. 10203351 10.1681/ASN.V104685

[20] Birn H, Fyfe JC, Jacobsen C, Mounier F, Verroust PJ, Orskov H, et al Cubilin is an albumin binding protein important for renal tubular albumin reabsorption. The Journal of clinical investigation. 2000;105(10):1353–61. Epub 2000/05/17. PubMed Central PMCID: PMC315466. doi: 10.1172/JCI8862 10811843 10.1172/JCI8862 PMC315466

[21] Storm T, Emma F, Verroust PJ, Hertz JM, Nielsen R, Christensen EI. A patient with cubilin deficiency. N Engl J Med. 2011;364(1):89–91. Epub 2011/01/07. doi: 10.1056/NEJMc1009804 21208123 10.1056/NEJMc1009804

[22] Hvidberg V, Jacobsen C, Strong RK, Cowland JB, Moestrup SK, Borregaard N. The endocytic receptor megalin binds the iron transporting neutrophil-gelatinase-associated lipocalin with high affinity and mediates its cellular uptake. FEBS letters. 2005;579(3):773–7. Epub 2005/01/27. doi: 10.1016/j.febslet.2004.12.031 15670845 10.1016/j.febslet.2004.12.031

[23] Kaseda R, Iino N, Hosojima M, Takeda T, Hosaka K, Kobayashi A, et al Megalin-mediated endocytosis of cystatin C in proximal tubule cells. Biochemical and biophysical research communications. 2007;357(4):1130–4. Epub 2007/04/28. doi: 10.1016/j.bbrc.2007.04.072 17462596 10.1016/j.bbrc.2007.04.072

[24] Charlton JR, Portilla D, Okusa MD. A basic science view of acute kidney injury biomarkers. Nephrology, dialysis, transplantation: official publication of the European Dialysis and Transplant Association—European Renal Association. 2014;29(7):1301–11. PubMed Central PMCID: PMCPMC4081632. 10.1093/ndt/gft510 PMC4081632 24385545

[25] Weyer K, Storm T, Shan J, Vainio S, Kozyraki R, Verroust PJ, et al Mouse model of proximal tubule endocytic dysfunction. Nephrology, dialysis, transplantation: official publication of the European Dialysis and Transplant Association—European Renal Association. 2011;26(11):3446–51. Epub 2011/09/20. 10.1093/ndt/gfr525 21926402

[26] Moestrup SK, Nielsen S, Andreasen P, Jorgensen KE, Nykjaer A, Roigaard H, et al Epithelial glycoprotein-330 mediates endocytosis of plasminogen activator-plasminogen activator inhibitor type-1 complexes. The Journal of biological chemistry. 1993;268(22):16564–70. Epub 1993/08/05. 8344937

[27] Nykjaer A, Fyfe JC, Kozyraki R, Leheste JR, Jacobsen C, Nielsen MS, et al Cubilin dysfunction causes abnormal metabolism of the steroid hormone 25(OH) vitamin D(3). Proceedings of the National Academy of Sciences of the United States of America. 2001;98(24):13895–900. Epub 2001/11/22. PubMed Central PMCID: PMC61138. doi: 10.1073/pnas.241516998 11717447 10.1073/pnas.241516998 PMC61138

[28] Cui S, Verroust PJ, Moestrup SK, Christensen EI. Megalin/gp330 mediates uptake of albumin in renal proximal tubule. The American journal of physiology. 1996;271(4 Pt 2):F900–7. Epub 1996/10/01. 8898021 10.1152/ajprenal.1996.271.4.F900

[29] Nielsen R, Christensen EI, Birn H. Megalin and cubilin in proximal tubule protein reabsorption: from experimental models to human disease. Kidney international. 2016;89(1):58–67. doi: 10.1016/j.kint.2015.11.007 26759048 10.1016/j.kint.2015.11.007

[30] Gburek J, Verroust PJ, Willnow TE, Fyfe JC, Nowacki W, Jacobsen C, et al Megalin and cubilin are endocytic receptors involved in renal clearance of hemoglobin. Journal of the American Society of Nephrology: JASN. 2002;13(2):423–30. Epub 2002/01/24. 11805171 10.1681/ASN.V132423

[31] Konopska B, Gburek J, Golab K, Warwas M. Characterization of chicken cystatin binding to rat renal brush-border membranes. Comparative biochemistry and physiology Part B, Biochemistry & molecular biology. 2007;146(4):482–8. Epub 2007/02/06. 10.1016/j.cbpb.2006.11.004 17275377

[32] Christensen EI, Gliemann J, Moestrup SK. Renal tubule gp330 is a calcium binding receptor for endocytic uptake of protein. The journal of histochemistry and cytochemistry: official journal of the Histochemistry Society. 1992;40(10):1481–90. Epub 1992/10/01. 1382088 10.1177/40.10.1382088

[33] Orlando RA, Rader K, Authier F, Yamazaki H, Posner BI, Bergeron JJ, et al Megalin is an endocytic receptor for insulin. Journal of the American Society of Nephrology: JASN. 1998;9(10):1759–66. Epub 1998/10/17. 9773776 10.1681/ASN.V9101759

[34] Togashi Y, Sakaguchi Y, Miyamoto M, Miyamoto Y. Urinary cystatin C as a biomarker for acute kidney injury and its immunohistochemical localization in kidney in the CDDP-treated rats. Experimental and toxicologic pathology: official journal of the Gesellschaft fur Toxikologische Pathologie. 2012;64(7–8):797–805. 21377848 10.1016/j.etp.2011.01.018

[35] Shanley PF, Rosen MD, Brezis M, Silva P, Epstein FH, Rosen S. Topography of focal proximal tubular necrosis after ischemia with reflow in the rat kidney. The American journal of pathology. 1986;122(3):462–8. PubMed Central PMCID: PMC1888206. 3953769 PMC1888206

[36] Schreiber A, Theilig F, Schweda F, Hocherl K. Acute endotoxemia in mice induces downregulation of megalin and cubilin in the kidney. Kidney international. 2012;82(1):53–9. doi: 10.1038/ki.2012.62 22437417 10.1038/ki.2012.62

[37] Kim HJ, Moradi H, Yuan J, Norris K, Vaziri ND. Renal mass reduction results in accumulation of lipids and dysregulation of lipid regulatory proteins in the remnant kidney. American journal of physiology Renal physiology. 2009;296(6):F1297–306. Epub 2009/04/10. PubMed Central PMCID: PMC2692452. doi: 10.1152/ajprenal.90761.2008 19357177 10.1152/ajprenal.90761.2008 PMC2692452

[38] Huby AC, Rastaldi MP, Caron K, Smithies O, Dussaule JC, Chatziantoniou C. Restoration of podocyte structure and improvement of chronic renal disease in transgenic mice overexpressing renin. PLoS One. 2009;4(8):e6721 Epub 2009/08/22. PubMed Central PMCID: PMC2725297. doi: 10.1371/journal.pone.0006721 19696925 10.1371/journal.pone.0006721 PMC2725297

[39] Tojo A, Onozato ML, Ha H, Kurihara H, Sakai T, Goto A, et al Reduced albumin reabsorption in the proximal tubule of early-stage diabetic rats. Histochemistry and cell biology. 2001;116(3):269–76. doi: 10.1007/s004180100317 11685557 10.1007/s004180100317

[40] Thelle K, Christensen EI, Vorum H, Orskov H, Birn H. Characterization of proteinuria and tubular protein uptake in a new model of oral L-lysine administration in rats. Kidney international. 2006;69(8):1333–40. Epub 2006/03/02. doi: 10.1038/sj.ki.5000272 16508656 10.1038/sj.ki.5000272

